# Factors influencing lion movements and habitat use in the western Serengeti ecosystem, Tanzania

**DOI:** 10.1038/s41598-022-22053-y

**Published:** 2022-11-07

**Authors:** Sarah L. Schooler, Shannon P. Finnegan, Nicholas L. Fowler, Kenneth F. Kellner, Ashley L. Lutto, Jamshid Parchizadeh, Merijn van den Bosch, Alejandra Zubiria Perez, Lusato M. Masinde, Stanslaus B. Mwampeta, Hailey M. Boone, Mariela G. Gantchoff, Jacob E. Hill, Todd M. Kautz, Nathaniel H. Wehr, Robert Fyumagwa, Jerrold L. Belant

**Affiliations:** 1 Department of Environmental Biology, State University of New York College of Environmental Science and Forestry, 1 Forestry Drive, Syracuse, NY 13210 USA; 2 Department of Fisheries and Wildlife, Michigan State University, East Lansing, MI 48824 USA; 3 Tanzania Wildlife Management Authority, Bariadi, Simiyu United Republic of Tanzania; 4 Department of Biology, University of Dayton, Dayton, OH 45469 USA; 5 Savannah River Ecology Laboratory, University of Georgia, Aiken, SC 29802 USA; 6 Tanzania Wildlife Research Institute, Arusha, United Republic of Tanzania

**Keywords:** Animal behaviour, Behavioural ecology, Conservation biology

## Abstract

Protected areas that restrict human activities can enhance wildlife habitat quality. Efficacy of protected areas can be improved with increased protection from illegal activities and presence of buffer protected areas that surround a core protected area. Habitat value of protected areas also can be affected by seasonal variation in anthropogenic pressures. We examined seasonal space use by African lions (*Panthera leo*) within a core protected area, Serengeti National Park, Tanzania, and surrounding buffer protected areas with varying protection strengths. We used lion locations in logistic regression models during wet and dry seasons to estimate probability of use in relation to protection strength, distance to protected area edge, human and livestock density, distance to roads and rivers, and land cover. Lions used strongly protected buffer areas over the core protected area and unprotected areas, and moved away from protected area boundaries toward the core protected area when buffer protected areas had less protection. Lions avoided high livestock density in the wet season and high human density in the dry season. Increased strength of protection can decrease edge effects on buffer areas and help maintain habitat quality of core protected areas for lions and other wildlife species.

## Introduction

Many mammal species are increasingly experiencing range contractions, population declines, and extirpations due to anthropogenic destruction of habitat, increased persecution, and overexploitation^1–3^. Large carnivores are especially affected because their extensive ranges and prey requirements often make them more susceptible to human-wildlife conflicts^4,5^, which have contributed to their population declines and range contractions during the past two centuries^2,4,6^. Protected areas that restrict human activities such as hunting, livestock grazing, logging, or land conversion are crucial for large carnivore conservation, as they can mediate anthropogenic pressures on wildlife because they conserve habitat^7^ and reduce human-wildlife conflicts^8^, and thus extinction risk^9^.

Carnivores experience higher levels of human-wildlife conflicts close to the edges of protected areas^8,10^. However, these “edge effects” can be mediated through the presence of buffer protected areas that surround a core protected area and buffer the core protected area from anthropogenic impacts, therefore enhancing the conservation value of the core protected area^11–14^. Though buffer areas sometimes permit hunting, livestock grazing, and other sustainable resource uses^11,12^, they simultaneously allow carnivores to reduce interactions with humans by moving further from populated edges into better quality core habitat^11,13,15^. However, fewer restrictions on resource use, inadequate law enforcement against illegal activities, and limited community-based benefit sharing (i.e., protected areas provide services or direct payments to surrounding communities) can lead to weaker protection of protected areas, and therefore may allow increased human and livestock incursions and poaching^16,17^. These human activities can reduce habitat quality and decrease effectiveness of protected areas for carnivores^16,17^.

Effectiveness of protected areas for wildlife may also vary seasonally due to variation of anthropogenic pressures or prey distribution^18–20^. To mediate anthropogenic pressures, carnivores within protected areas can alter their habitat use seasonally to avoid interactions with humans^21^. Carnivores may avoid areas closer to roads or with higher human populations during periods of higher tourism, legal hunting, or poaching^20^. During times of year with reduced forage, there may be increased livestock incursions into protected areas, where there is better forage^18,19^, which may in turn shift carnivores away from protected area edges, especially in protected areas with limited law enforcement^22^. Alternatively, during times of resource scarcity, carnivores may be attracted to protected area edges due to availability of livestock as alternative prey, which can increase human-carnivore conflicts^23,24^. Non-anthropogenic factors such as water availability and land cover can influence prey distribution seasonally, which in turn can alter carnivore habitat use^18,25^. During periods of resource scarcity, prey congregate closer to limited resources^18,25^, and carnivore habitat use shifts to follow prey distribution^20,26,27^.

African lions (*Panthera leo*) are especially susceptible to human-wildlife conflicts due to their large home ranges and potential threat to humans and their livelihoods^28^. Lions within protected areas experience a gradient of seasonal negative interactions with humans, ranging from human and livestock incursions to poaching and bushmeat hunting, due in part to varying protection among protected areas^29^. Because of lions’ susceptibility to human-wildlife conflicts and the seasonal variation in this susceptibility, it is important to determine the impact that varying protection strength and presence of buffer protected areas have on lion space use, while incorporating a gradient of potential human interactions, water availability, and land cover types.

**Figure 1:**
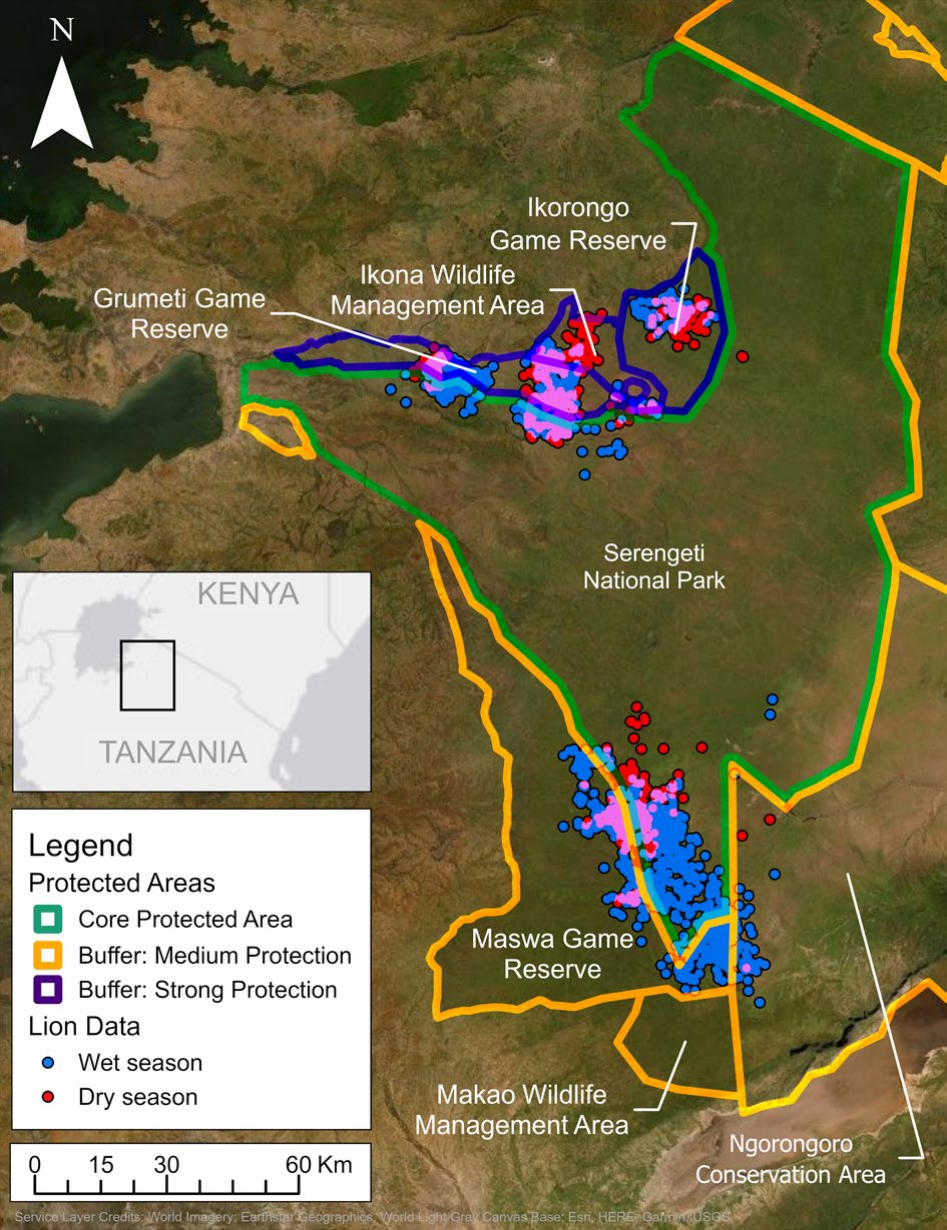
Locations of GPS-collared lions during the wet (November–May) and dry (June–October) seasons in the northern and southern study areas in the protected area complex, with protected area classification (core protected area, buffer protected areas with medium or strong protection)^38^, Serengeti ecosystem, Tanzania, 2018–2019. Map was created using ESRI ArcGIS Pro Version 2.9 using World Imagery basemap, 2022, Esri Inc [https://www.arcgis.com/home/item.html?id=10df2279f9684e4a9f6a7f08febac2a9].

The Serengeti ecosystem is in northwest Tanzania and is comprised of Serengeti National Park (SNP) and a surrounding network of game reserves and conservation areas, which together create a protected area complex (Fig. 1)^19,30^. The surrounding game reserves and conservation areas act as a buffer (“buffer protected areas”) to reduce cross-boundary human pressures on the core protected area (SNP)^12,19,29–31^. Because the surrounding buffer areas have varying regulations and amounts of funding, law enforcement, and community-based benefit sharing^12,19,32^, this protected area complex is an ideal system to examine variation in effectiveness of buffer protected areas for lions. Though much is known about lion habitat use in the Serengeti ecosystem^27,33,34^, there has been little research on how buffer protected areas and their protection strength influence lion space use^35^.

Our purpose was to determine how strength of protection of protected areas, presence of buffer areas, human and livestock presence, roads, water sources, and land cover affect lion seasonal space use. We predicted that lions would disproportionately use areas with stronger protection and further toward the interior of the protected area complex, and their habitat use would vary seasonally due to differences in prey distribution and human activities. We also predicted that lions would select for areas with lower human population and livestock density and distant from roads, and that these effects would be greater in the dry season. Because prey congregate closer to water and move into shaded areas with thicker, denser forage during periods of resource scarcity^18,25^, we predicted that lions would disproportionately use these areas during the dry season.

## Methods

### Study area

This study was conducted in northern and southern portions of the western corridor of SNP, Tanzania, and surrounding protected areas which together comprise a "protected area complex" (2° S, 35° W, 40,000 km^2^; Fig. 1). Serengeti National Park has the highest conservation priority within this protected area complex and therefore we refer to it as the core protected area in the Serengeti ecosystem^29–31^. We defined buffer areas as areas within the protected area complex that surround the core protected area of SNP, though we recognize that some of the protected areas we classified as buffer areas could be considered core protected areas^19^.

The northern study area (3500 km^2^) included parts of northern SNP and buffer protected areas of Grumeti Game Reserve (GGR), Ikona Wildlife Management Area (IWMA), and Ikorongo Game Reserve (IGR), along with adjacent unprotected areas (Fig. 1). The southern study area (3300 km^2^) included parts of southern SNP, and buffer areas Ngorongoro Conservation Area (NCA; designated World Heritage Site) and Maswa Game Reserve (MGR; designated IUCN category IV [habitat/species management area])^30^. Makao Wildlife Management Area is also part of the protected area complex, but lions in our study did not use this protected area and therefore we excluded it from analysis. There are no anthropogenic barriers in the protected area complex except for a 30-km fence along a portion of the northern border of GGR that is unlikely to restrict lion movement^36^. Serengeti National Park and surrounding game reserves and wildlife management areas prohibit livestock grazing and agriculture, and largely prohibit human settlement, while NCA allows human settlement, sustainable resource extraction, and regulated livestock grazing^32,37^. Lion harvest is prohibited in Tanzania except in game reserves and wildlife management areas, but GGR, IWMA, and IGR prohibit hunting^31^. Harvest of lions is allowed in MGR during 1 July–31 December, but no legal harvest of lions has occurred in MGR since 2013^35^. The majority of tourism activity occurs in the Serengeti protected area complex during May–September^39^.

Annual rainfall varies along a southeast (500 mm) to northwest (1100 mm) gradient, with rain typically occurring from November to May^40^. The study area contains sparse woodland-grassland with patches of dense woodland, as well as some cultivated areas^19,41^. There is a seasonal migration of about 1.3 million wildebeest (*Connochaetes taurinus*) across the ecosystem^40,43^. During most of the wet season (December–April), wildebeest occur throughout the southern part of our study area before migrating north through the western corridor during the early dry season, and crossing through the northern portion of our study area during August–November^43^ (Supplementary Fig. S1).

### Data collection

We captured 16 lions (12 female, 4 male) from 11 prides during March–November 2018 using broadcasted vocalizations^44^ and rifle-fired (Palmer CapChur SS cartridge-fired rifle; Cap-Chur Equipment, Powder Springs, Georgia, USA) darts (Pneudart Type U Remote Delivery Devices; Pneudart Inc., Williamsport, PA, USA) from vehicles^45^. We equipped lions with global positioning system (GPS) collars (Model IR-SAT, African Wildlife Tracking, South Africa) programmed to obtain hourly locations. Animal capture and handling protocols for conducting darting and collaring were approved by State University of New York College of Environmental Science and Forestry (180502) Institutional Animal Care and Use Committee. Conduct of research was approved and permitted by the Tanzania Commission for Science and Technology (2018-6-ER-2016-125) and the Tanzanian Wildlife Research Institute. This study was carried out in compliance with ARRIVE guidelines and all methods were carried out in accordance with relevant guidelines and regulations.

We used previously published strength of border control to represent strength of protection for buffer protected areas^19^. The classification was based qualitatively on amount of funding, consistency of border patrols and other law enforcement, prevalence of illegal activity, and level of community-based benefit sharing (Fig. 1; Supplementary Methods)^12,19,29,31^. Grumeti Game Reserve, IGR, and IWMA were categorized as strongly protected buffer areas, while MGR and NCA were categorized as having medium protection (Fig. 1)^38^. Grumeti Game Reserve, IGR, and IWMA are managed jointly by the government of Tanzania and Singita Grumeti Limited, a private company which strictly limits trophy hunting^31^, and provides law enforcement resources^19,46–49^ and increased community-based benefit sharing^31,49–52^. Thus, GGR, IGR, and IWMA experience less poaching^12,46,53^, livestock incursion^19,46^, and illegal crop cultivation^19,54^ and timber harvest^46^ as compared to MGR and NCA^19,29,55^.

To account for influences of land cover on lion space use, we used data from Copernicus Global Land Operations level-1 land cover classification, combining open and closed forest land cover types^42^. Our final land cover categories were cultivated land, herbaceous vegetation, shrublands, and forest^42^. We calculated distance from roads and rivers using data from Serengeti GIS and Data Centre, removing rivers classified as ephemeral^56,57^. We obtained gridded 2018 human population density (people/hectare) from Worldpop^58^. We summed number of cattle, sheep and goats from the 2010 Gridded Livestock of the World Database (9.26 km^2^ resolution) to estimate total livestock density (individuals/9.26 km^2^)^59–62^.

### Analysis

We removed GPS location data for five days following capture to account for potential capture effects. We randomly thinned hourly lion GPS locations to one location each day per lion (“used” points) to reduce spatial and temporal autocorrelation^63^. We separated lion GPS locations between the north and south study areas and created separate 95% kernel density estimates around used points in each area using the R package “adehabitat”^64–66^. We then generated an equal number of random available locations within each 95% kernel and combined points across the two areas for our complete dataset.

We extracted distance inside protected area complex (points outside the complex had a negative value), protected area classification (buffer area with strong or medium protection, core protected area, or unprotected area), human and cattle densities, distance to nearest road, distance to nearest river, and land cover classification for each used and available point using program R^66^. We normalized continuous variables (distance-based metrics and human and cattle densities), then calculated pairwise Pearson product-moment correlations, finding no strong correlations (|*r*|< 0.7)^67^. We separated data into wet (November–May) and dry (June–October) seasons^33^, then used logistic regression models for each season to determine the effects of level of protection, distance to protected area edges, human and livestock density, distance to rivers and roads, and land cover on lion probability of use^68^. Because lion used and available points in the northern study area were within buffer protected areas with strong protection, the core protected area, and unprotected areas, whereas lion points in the southern area were within buffer protected areas with medium protection and the core protected area, we included an interaction factor between area (north and south) and distance to protected area edge to test the effect of buffers while accounting for the differences in protected area strength. To determine the effects of seasonality on lion probability of use, we fit separate models for wet and dry seasons.

We tested the predictive accuracy of our models with k-fold cross-validation using five folds to calculate area under the receiver operating characteristic curve (AUC)^69^. We used the statistical significance of individual covariate effects using Wald tests with α = 0.05 to determine which variables contributed to lion probability of use for each season^70,71^. We compared directionality and effect size of each significant (p < 0.05) variable between seasons to determine which variables most affected lion probability of use. We also computed estimated marginal means for both models to determine significant differences between the effects of protected area classification and land cover type using R package “emmeans”^72^. We used program R for all analyses^66^.

## Results

Our final dataset included 3612 locations from 16 lions (12 female, 4 male) with an average of 226 locations per lion (range = 79–400, Supplementary Table S1). The sex ratio of collared lions was a consequence of lion availability during captures, but broadly represented the adult sex structure in the population^73^. We obtained 2418 locations during the wet season and 1194 locations during the dry season. Both seasonal models had adequate fit based on K-fold cross validation (wet season AUC = 0.64, dry season AUC = 0.76).

**Figure 2:**
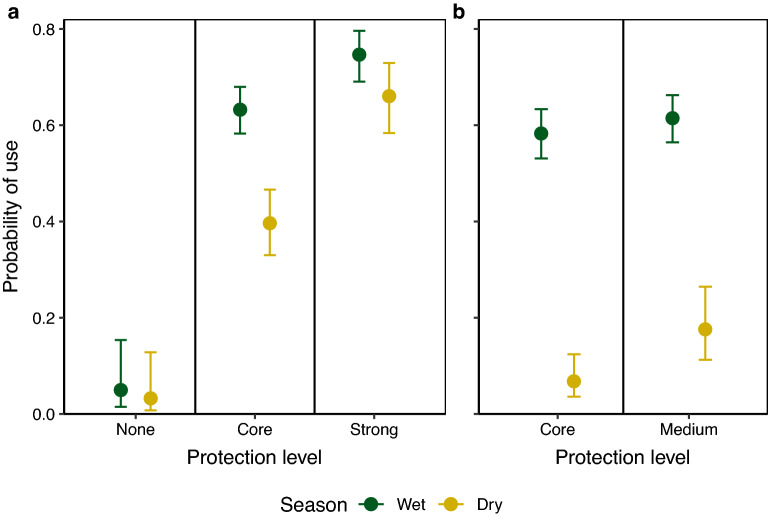
Lion probability of use and 95% confidence intervals in areas without protected status (“none”), core protected areas (“core”), buffer protected areas with medium protection (“medium”), and buffer protected areas with strong protection ("strong”)^38^ during wet (November–May) and dry (June–October) seasons in the northern (**a**) and southern (**b**) study areas, holding other continuous variables constant at mean values and using categorical variables of female sex and cultivated land, Serengeti ecosystem, Tanzania, 2018–2019.

**Figure 3:**
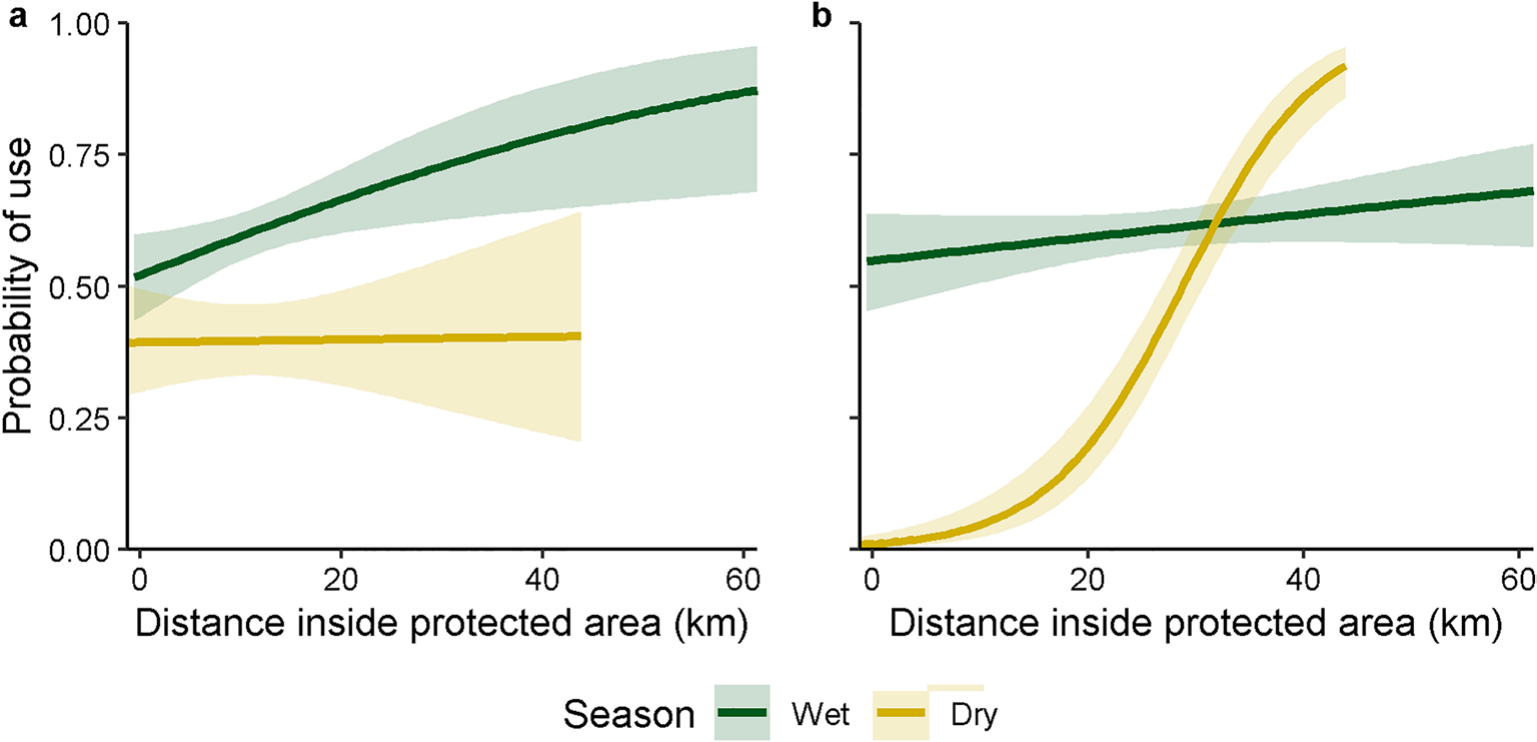
Lion probability of use and 95% confidence intervals relative to distance from edge of the protected area complex (km) in northern (**a**) and southern (**b**) study areas during wet (November–May) and dry (June–October) seasons, holding other continuous variables constant at mean values and using categorical variables of female sex, core protection, and cultivated land, Serengeti ecosystem, Tanzania, 2018–2019.

In the northern study area, lion probability of use of buffer protected areas with strong protection and the core protected area was greater than probability of use of unprotected areas, particularly during the wet season (Table 1, Fig. 2, Supplementary Table S2). Lion probability of use was similar in the core protected area and buffer areas with medium protection in the southern study area during the wet season, but in the dry season probability of use of buffer areas with medium protection was greater than probability of use of the core protected area. Lion probability of use increased with increasing distance inside the protected area complex in the wet season in both study areas, but during the dry season, lion probability of use increased with increasing distance inside protected area complex borders only in the southern study area (Fig. 3).

**Table 1:** Parameter estimates, standard errors (SE), and p-values for lion probability of use during the wet (November–May) and dry (June–October) seasons, Serengeti ecosystem, Tanzania, 2018–2019. Values for categorical variables are as compared to reference categories of cultivated (land cover), no protection (protected area status), female (sex), and the northern study area. Significant predictors (p < 0.05) are marked in bold.

Parameter	Wet season			Dry season		
	Estimate	SE	p-value	Estimate	SE	p-value
**Land cover**						
Herbaceous	**− 0.651**	**0.076**	**< 0.001**	**− 0.712**	**0.121**	**< 0.001**
Shrublands	**− **0.048	0.080	0.547	**− **0.043	0.132	0.744
Forest	**− 0.402**	**0.138**	**0.004**	0.333	0.199	0.095
Population density (people/hectare)	**0.220**	**0.045**	**< 0.001**	**− 0.171**	**0.071**	**0.016**
Livestock density (livestock/9.26 km^2^)	**− 0.248**	**0.051**	**< 0.001**	**− **0.139	0.081	0.087
Distance from roads (km)	**− 0.281**	**0.035**	**< 0.001**	**− 0.276**	**0.055**	**< 0.001**
Distance from rivers (km)	**− **0.059	0.034	0.085	**− 0.577**	**0.06**	**< 0.001**
Distance inside protected areas (km)	**0.311**	**0.122**	**0.011**	0.011	0.142	0.939
**Protected area status**						
Core	**3.490**	**0.629**	**< 0.001**	**2.971**	**0.753**	**< 0.001**
Medium	**3.622**	**0.637**	**< 0.001**	**4.050**	**0.783**	**< 0.001**
Strong	**4.029**	**0.615**	**< 0.001**	**4.058**	**0.731**	**< 0.001**
**Sex**						
Male	0.121	0.079	0.124	0.030	0.117	0.798
**Study area**						
South	**− **0.208	0.135	0.122	**− 2.201**	**0.340**	**< 0.001**
**Area × distance inside protected areas**						
South × distance (km)	**− **0.216	0.135	0.110	**1.576**	**0.225**	**< 0.001**

**Figure 4:**
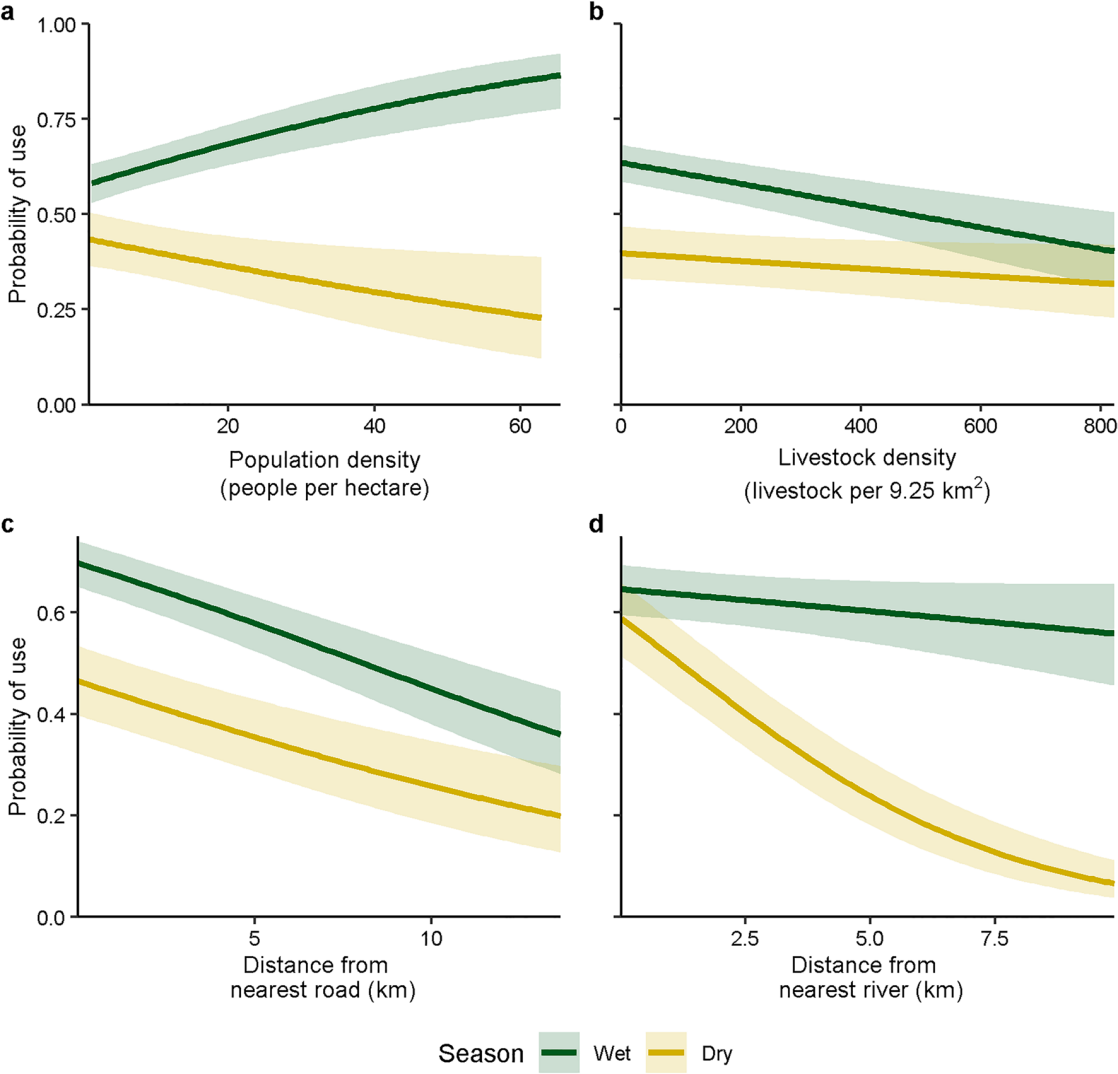
Lion probability of use and 95% confidence intervals relative to (**a**) population density (people/hectare), (**b**) livestock density (livestock/9.26 km^2^), (**c**) distance from nearest road (km), and (**d**) distance from nearest river (km) during wet (November–May) and dry (June–October) seasons, holding all other continuous variables constant at mean values, and using categorical variables of female sex, northern area, core protection, and cultivated land cover, Serengeti ecosystem, Tanzania, 2018–2019.

**Figure 5:**
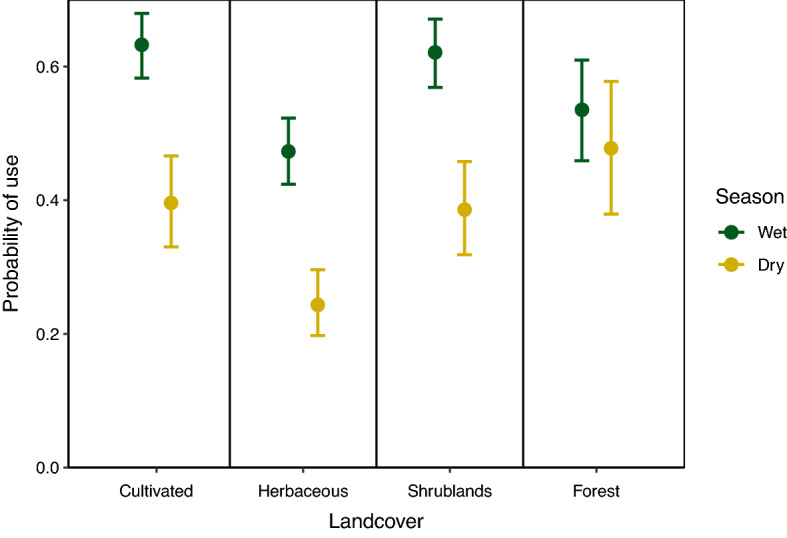
Lion probability of use and 95% confidence intervals in cultivated lands, herbaceous vegetation, shrublands, and forest during wet (November–May) and dry (June–October) seasons, holding other continuous variables at mean values and using categorical variables of female sex, northern area, and core protection, Serengeti ecosystem, Tanzania, 2018–2019.

During the wet season, lion probability of use increased with increasing human population density and decreased with increasing livestock density and distance from roads (Table 1, Fig. 4). During the dry season, lion probability of use decreased with increasing human population density, distance from roads, and distance from rivers. Lion probability of use during the wet season was greatest in cultivated areas and shrublands, intermediate in forest, and lowest in herbaceous vegetation (Fig. 5; Supplementary Table S3). Lion probability of use during the dry season was greatest in forest land cover, intermediate in cultivated areas and shrublands, and lowest in herbaceous vegetation.

## Discussion

Lions demonstrated lower probability of use of unprotected areas as compared to core protected areas and strongly protected buffer areas, which confirms the importance of protected areas to conserve lion habitat^17,74^. In the northern study area, lions selected for strongly protected buffer areas (GGR, IWMA, and IGR) over non-protected and core protected areas. In the southern study area, lions used buffer areas with medium protection and the core protected area similarly in the wet season, whereas in the dry season, lions had higher probability of use in buffer areas with medium protection as compared to the core protected area.

Though lion probability of use increased similarly in the north and the south with increasing distance to protected area edges in the wet season, lions avoided protected area edges in the dry season only in the southern study area, where buffer areas have medium protection. In the dry season human and livestock incursions into protected areas increase because forage quality is better^18,19,75,76^ and water is more plentiful^31^ inside protected areas. Additionally, there is increased poaching in the dry season due to food scarcity^77^. That lions avoided protected area edges in the dry season only in buffer areas with medium protection rather than strong protection indicates that increased protection strength decreases impacts of edge effects on lions, especially during periods of resource scarcity.

Strongly protected buffer areas have better law enforcement and increased community-based benefit sharing which decrease prevalence of illegal grazing and poaching^29,37,78^. These measures can result in decreased edge effects^19^ including human disturbance^21^ and increased prey availability^17,79^, resulting in higher lion survival^80^ and abundance^17^. Buffer areas with stronger protection may provide high quality habitat within their borders and therefore may function as core protected areas^19,81^. We suggest that because buffer protected areas with weaker protection experience increased edge effects^19^, their edges provide lower quality habitat than edges of strongly protected buffer areas. However, we found that toward the interior of the protected area complex, buffer areas with medium protection provided similar habitat value for lions as compared to core protected areas, while allowing lions to move away from protected area complex edges and increasing habitat value of the core protected area^35^.

Though seasonal avoidance of protected area complex edges is likely due to greater anthropogenic pressures^19,35,82^, lion avoidance of edges may also have been influenced by seasonal prey distributions^26,43^. During the dry season in the northern study area, increased lion use of strongly protected buffer areas over the core protected area was more pronounced and lions did not avoid protected area edges, as compared to the wet season. This pattern may be due to wildebeest migrating through the northern strongly protected areas during August–October (dry season; Supplementary Fig. S1); this increased prey availability in the strongly protected areas and closer to the edges of the protected area complex likely attracts lions into those areas^43^. In contrast, lions in the southern study area continued to avoid protected area edges when wildebeest were present (in the wet season), though wildebeest occur throughout the buffer protected areas, providing increased prey accessability^39,43^. Additionally, when wildebeest were not present (dry season in the south; wet season in the north), lions demonstrated greater avoidance of protected area edges in the less strongly protected southern study area as compared to the more strongly protected northern study area. Our findings indicate that while wildebeest presence influences lion habitat use^33^, strength of protection for buffer protected areas also contributes to lion habitat use.

We found that lions disproportionately used forested areas more than other land cover types in the dry season, while in the wet season they used cultivated areas and shrublands more than herbaceous land cover or forest. While wild ungulates are widely dispersed across the landscape in the wet season, they frequently congregate near permanent water in the dry season^18,25^. Permanent water sources not only provide water, but the associated woodlands provide a foraging refuge as well as shade^22,23,25,26^. Therefore, that lions had increased probability of use of forested land cover and closer to permanent water sources in the dry season was likely due to location of prey as well as water availability^22,83,84^, whereas increased probability of use of cultivated areas and shrublands in the wet season may have been due to more dispersed prey^18,20,26^.

Lion probability of use decreased with increasing distance to roads, potentially because roads facilitate stealth predation^27^ or because lions, like other large carnivores^85^, use roads to facilitate travel^86^. Additionally, that lions did not avoid roads in either season indicates that photographic tourism may not strongly influence lion space use in this ecosystem because lions in this area are habituated to vehicles^17,87^. Lion probability of use was negatively related to livestock density during the wet season. Increased livestock presence can reduce effectiveness of protected areas for lions because livestock are associated with increased human presence and therefore human-wildlife conflicts^17^. Livestock also may compete for resources with wild ungulates, potentially reducing prey availability for lions^11,18^. That lions only avoided livestock during the wet season, and that lion habitat use was influenced by distance to nearest river only in the dry season, suggests that decreased availability of water during the dry season concentrates humans and livestock at permanent water sources used by lions and their prey^23^. Therefore, water scarcity could lead to increased predation of livestock and increased human-wildlife conflicts^23,88^.

Consistent with our predictions, lion probability of use decreased with increasing human population density, but only in the dry season. Lions may have avoided areas of higher human population density especially during the dry season because more tourism, legal hunting, and poaching occur during this time^20,39,46,48^. In contrast to previous research^22,26,89^, we found that lion probability of use increased with increasing human population density in the wet season. We suggest that because prey are more widely distributed in the wet season^18,22,33^, lions may have used areas with higher human population due to difficulty of hunting wild prey^33^. Alternatively, because we were unable to account for seasonal variation in human population density, the areas lions used may have only appeared to have higher human population density, when in actuality the areas were sparsely inhabited. Human population density varies seasonally in the Serengeti ecosystem^90^, due to pastoral practices^22^ and increased use of tourist camps in the dry season^39^. We suggest that future research on lion habitat use considers seasonal variation in spatial distribution of humans.

Due to a lack of reliable data, we were unable to account for wild prey abundance or distribution. Together with anthropogenic activity^91^, prey availability is a major determinant of lion space use^20,26,27^. Therefore, increased lion probability of use of areas with stronger protection was likely driven not only by the wildebeest migration, but also non-migratory prey availability in these areas, as both lions and their prey benefit from lower human disturbance provided by protected areas^17^. Our findings on lion use of buffer areas were influenced by migrating wildebeest, but by separating northern and southern study areas and modeling seasons separately we were able to incorporate the effects of wildebeest migration on lion space use^43^. Additionally, though we collared lions from 11 prides, lion space use could have been influenced by territoriality of surrounding lion prides, and increased lion use of buffer protected areas with medium protection as compared to core protected areas may be due to territoriality of lion prides within the core protected area^34^. Lion lack of use of Makao Wildlife Management Area may have been due to lion territoriality or where lions were captured. Similarly, we only used data from lions collared in buffer protected areas, so our results are applicable only to lions that primarily use these areas.

An additional caveat is that the classification of strength of protection in protected areas we used is inherently qualitative. Many factors can influence the effectiveness of protected areas for wildlife including adjacent human density^7,19^, community support of protected areas^7^, extent of community involvement^92^, corruption in protected area management^29^, amount and efficacy of law enforcement^10^, and economic conditions of the surrounding communities^92^ and country^10^. The protected areas in this study had varying combinations of these factors^31,32,46^, and therefore their efficacy for protecting wildlife likely varied within categories. However, the categorization of buffer protected areas into areas with medium and strong protection is supported quantitatively^19,46^, with areas categorized as medium protection having more livestock incursions, illegal agriculture, poaching, and timber harvest than areas that were strongly protected (Supplementary Methods)^19,46,47^. Improved quantification of law enforcement, community-based benefit sharing, and protected area funding would be beneficial to determine which factors of protected area strength are most influential for lion habitat use and would contribute to protected area management.

Creation of effective protected areas for large carnivore conservation requires an understanding of seasonal factors that affect their habitat use^21,89^. Our findings on seasonal lion habitat use in relation to human and livestock density, distance to roads and rivers, and land cover were broadly consistent with previous research that demonstrated that lions balance seasonal patterns of prey availability^22,33^ with avoiding humans^20,93^. We also quantified the importance of buffer protected areas and their strength of protection for lions. Buffer protected areas likely increase habitat value of core protected areas for large carnivores by allowing them to move away from human disturbance along the edges of buffer protected areas into core protected areas^35^. Additionally, buffer protected areas that provide increased funding and alternative employment for neighboring communities and have increased enforcement against illegal activity not only enhance integrity of core protected areas, but also themselves may provide high quality habitat. While strongly protected buffer areas may increase suitable habitat, those with less protection can reduce edge effects for core protected areas and therefore maintain quality of wildlife habitat within core protected areas.

## Supplementary Information

Supplementary Information.

## Data Availability

Raw data on animal locations are unavailable to ensure the well-being of the animals. All other datasets used during the current study are available from the corresponding author on reasonable request.
