# Supplementary material for: Factors influencing lion movements and habitat use in the western Serengeti ecosystem, Tanzania
Source: Sci Rep. 2022 Nov 7;12:18890. doi: 10.1038/s41598-022-22053-y (PMC9640537; doi:10.1038/s41598-022-22053-y)
Supplement: Supplementary file 1 — Supplementary Information. [file 41598_2022_22053_MOESM1_ESM.pdf]

Supplementary Information for "Factors influencing lion movements and habitat use in the western Serengeti ecosystem, Tanzania"

Authors: Sarah L. Schooler[1,2*], Shannon P. Finnegan[1], Nicholas L. Fowler[1], Kenneth F. Kellner[2], Ashley L. Lutto[1], Jamshid Parchizadeh[2], Merijn van den Bosch[2], Alejandra Zubiria Perez[2], Lusato M. Masinde[3], Stanslaus B. Mwampeta[3], Hailey M. Boone[2], Mariela G. Gantchoff[2], Jacob E. Hill[1] , Todd M. Kautz[2], Nathaniel H. Wehr[2], Robert Fyumagwa[3], Jerrold L. Belant[2]

[1] Department of Environmental Biology, State University of New York College of Environmental Science and Forestry, 1 Forestry Drive, Syracuse, New York, 13210 USA.
[2] Department of Fisheries and Wildlife, Michigan State University, East Lansing, Michigan, United States of America
[3] Tanzania Wildlife Management Authority, P.O Box 277, Bariadi, Simiyu, United Republic of Tanzania.
[4] Tanzania Wildlife Research Institute, PO Box 661, Arusha, United Republic of Tanzania.

* Corresponding author: Sarah L. Schooler, Global Wildlife Conservation Center, State University of New York College of Environmental Science and Forestry, 1 Forestry Drive, Syracuse, New York, 13210 USA. Email: sarahlschooler@gmail.com. Phone: 001 608 698 4770

Supplementary Table S1. Identification number (Lion ID), area (north [N] or south [S]), sex (male [M] or female [F], number of locations used for analysis during wet (November–May; n wet) and dry (June–October; n dry) seasons, and start and end date of collected locations for 16 GPS-collared lions, Serengeti ecosystem, Tanzania, 2018–2019.

Lion ID	Area	Sex	n wet	n dry	Start	End
BR_01	N	F	139	117	7/23/2018	9/19/2019
BR_02	N	F	155	0	11/5/2018	4/18/2019
BW_01	N	F	142	136	6/10/2018	3/22/2019
KR_01	N	F	253	147	3/29/2018	5/13/2019
ML_80	N	M	117	144	3/23/2018	12/17/2018
ML_81	N	M	218	144	4/16/2018	4/21/2019
RB_01	N	F	65	138	5/18/2018	12/21/2018
LS_01	S	F	193	1	11/7/2018	6/1/2019
MB_01	S	F	203	119	9/27/2018	8/30/2019
ML_90	S	M	107	36	9/26/2018	2/15/2019
ML_93	S	M	79	0	11/12/2018	1/29/2019
MO_01	S	F	168	0	11/13/2018	5/9/2019
SD_01	S	F	203	73	9/23/2018	7/7/2019
SI_01	S	F	103	6	9/26/2018	3/21/2019
SK_01	S	F	203	61	9/26/2018	6/26/2019
SM_01	S	F	70	72	5/10/2018	12/21/2018
Sum			2418	1194	3/23/2018	9/19/2019

Supplementary Table S2. Comparison of lion estimated marginal mean probability of use (estimate) in the core protected area (Serengeti National Park), strongly protected buffer areas, buffer areas with medium protection, and areas without protection, with season, area (north [N] or south [S]), standard error (SE) and p-values, Serengeti ecosystem, Tanzania, 2018–2019. Comparisons with significant differences ($p < 0.05$) are in bold type.

	Area	Comparison	Estimate	SE	p-value
Wet Season	N	**Strongly protected buffer: core protected area**	**0.539**	**0.123**	**<0.001**
	N	**Strongly protected buffer: no protection**	**4.029**	**0.615**	**<0.001**
	N	**Core protected area: no protection**	**3.490**	**0.629**	**<0.001**
	S	Medium protected buffer: core protected area	0.132	0.109	0.929
Dry Season	N	**Strongly protected buffer: core protected area**	**1.086**	**0.153**	**<0.001**
	N	**Strongly protected buffer: no protection**	**4.058**	**0.731**	**<0.001**
	N	**Core protected area: no protection**	**2.971**	**0.753**	**0.002**
	S	**Medium protected buffer: core protected area**	**1.079**	**0.226**	**<0.001**

Supplementary Table S3. Comparison of lion estimated marginal mean probability of use (estimate) in cultivated, herbaceous, shrubland, and forest land cover types, with season, standard error (SE) and p-values, Serengeti ecosystem, Tanzania, 2018–2019. Comparisons with significant differences ($p < 0.05$) are in bold type.

	Comparison	Estimate	SE	p-value
Wet Season	**Cultivated: herbaceous**	**0.651**	**0.077**	**<0.001**
	Cultivated: shrublands	0.048	0.080	0.932
	**Cultivated: forest**	**0.402**	**0.138**	**0.019**
	**Herbaceous: shrublands**	**-0.602**	**0.079**	**<0.001**
	Herbaceous: forest	-0.249	0.136	0.258
	Shrublands: forest	0.354	0.139	0.054
Dry Season	**Cultivated: herbaceous**	**0.712**	**0.121**	**<0.001**
	Cultivated: shrublands	0.043	0.132	0.988
	Cultivated: forest	-0.333	0.199	0.341
	**Herbaceous: shrublands**	**-0.669**	**0.115**	**<0.001**
	**Herbaceous: forest**	**-1.045**	**0.189**	**<0.001**
	Shrublands: forest	-0.376	0.194	0.214

Supplementary Figure S1. Seasonal pattern of wildebeest migration (black arrows) through the Serengeti ecosystem[1–3] with protected area boundaries (green polygon), Kenya and Tanzania. Map was created using ESRI ArcGIS Pro Version 2.9 using the Light Gray Basemap, 2022, Esri Inc [https://doc.arcgis.com/en/data-appliance/2022/maps/world-light-gray-base.htm]; wildebeest image adapted by author from public domain image by Pearson Scott Foresman [https://commons.wikimedia.org/wiki/File:Gnu_1_(PSF).png].

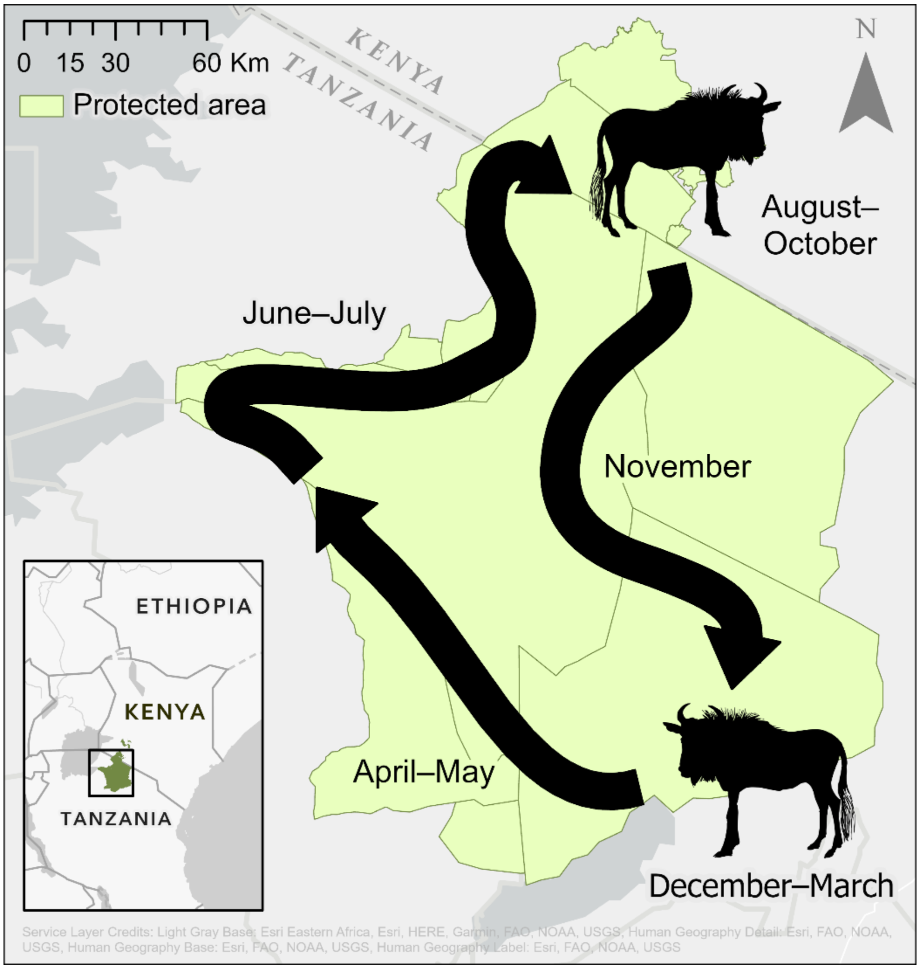

1. Larsen, F. et al. Wildebeest migration drives tourism demand in the Serengeti. Biol. Conserv. 248, 108688 (2020).
2. Thirgood, S. et al. Can parks protect migratory ungulates? The case of the Serengeti wildebeest. Anim. Conserv. 7, 113–120 (2004).
3. Boone, R. B., Thirgood, S. J. & Hopcraft, J. G. C. Serengeti wildebeest migratory patterns modeled from rainfall and new vegetation growth. Ecology 87, 1987–1994 (2006).

Supplementary Methods

These methods further describe the classification description of protection strength of buffer protected areas, Serengeti protected area complex, Tanzania. We used the classification scheme of Veldhuis et al. (2018, 2019) for buffer protected area strength of protection[1,2]. We further describe variation in protected area classification supporting this classification including protected area status both internationally and within Tanzania, legally permitted activities, community-based benefit sharing, community involvement in decision making, funding and level of law enforcement, and extent of illegal activities for the buffer protected areas in our study (Ngorongoro Conservation Area [NCA], Maswa Game Reserve [MGR], Grumeti Game Reserve [GGR], Ikorongo Game Reserve [IGR], and Ikona Wildlife Management Area [IWMA]).

Ngorongoro Conservation Area – medium protection

Ngorongoro Conservation Area (NCA) is managed by the Ngorongoro Conservation Area Authority (NCAA) and is categorized as a National Monument (IUCN Category III) and a UNESCO world heritage site[3,4]. The primary management objectives of the area are to conserve natural resources, protect the interests of the Maasai pastoralists, protect archeological sites, and promote tourism[2,3]. Sustainable resource use such as livestock grazing and tree cutting are permitted[4]. Though there are restrictions on livestock numbers and grazing in NCA, there were approximately 300,000 domestic livestock in 2017, and there is increasing demand by residents for subsistence crop cultivation[4]. Additionally, the resident population in NCA during 2017 was 93,136, leading to competition for resources with wildlife[4]. High tourism within NCA also may disrupt wildlife[4].

Ngorongoro Conservation Area Authority provides community-based benefit sharing through construction of schools and health centers, scholarships for local students, food and water for domestic use or livestock, access to forest products, and employment[5,6]. Local people also benefit economically from tourism, though they receive little to no direct payments from NCAA[4,7]. Local people perceive that they receive minimal benefits compared to what NCAA generates[8]. While protecting the interests and rights of the local people is a principal objective for NCA, there are few opportunities for local communities to engage in conservation[4,6,8].

Though hunting is prohibited in NCA, commercial and subsistence poaching as well as ritual and retaliatory killing occurs[9,10]. Recent information about poaching or law enforcement levels in NCA is unavailable, but there likely is political interference with anti-poaching operations in this area, leading to corruption and funding limitations[11,12]. Because human habitation and livestock are allowed, there is increased livestock grazing, burning, and human dwellings as well as lower vegetation productivity within NCA as compared to GGR, IGR, or IWMA[1,2].

Maswa Game Reserve – medium protection

Maswa Game Reserve (MGR) is managed by the Tanzania Wildlife Management Authority (TAWA) and is categorized as a nature conservation reserve (IUCN Category IV), which means that the area is managed for landscape conservation and recreation[3]. In Tanzania, game reserve status implies that occupation, livestock grazing, and cropland are prohibited, while trophy hunting is permitted[2,13]. Harvest of lions is allowed in MGR during 1 July–31 December, but no lions have been legally harvested since 2013[14].

Tanzania Wildlife Management Authority provides social services to communities by constructing schools, health centers, and roads as well as providing bush meat[5]. Additionally, TAWA partners with hunting companies to provide livelihood benefits to communities through legal hunting, which may provide limited employment opportunities, though TAWA provides no direct household payments or employment opportunities[5,6]. However, people living near MGR benefitted less from hunting and tourism revenue than people near other protected areas in the SNP protected area complex[13], potentially because this area experiences high levels of corruption[12] and lower levels of funding[15] as compared to other game reserves. Most decisions such as fines, protected area boundaries, and investments are made by the Tanzania Wildlife Division district councils with limited consultation of stakeholders[6].

Recent information about law enforcement levels in MGR is unavailable, but a greater number of wildlife officers have been killed in MGR than in elsewhere in the SNP protected area complex[12], with implications that politicians are reticent to fund law enforcement for protected areas or prosecute illegal activity[6,12,16]. Therefore, there is considerable encroachment for agriculture, mining, settlement, and grazing in MGR[2,9,12,15]. There is more area burned and decreased forage productivity in MGR as compared to GGR, IGR, and IWMA[1,2]. Additionally, more poaching occurs in MGR than in the north-west buffer protected areas (GGR, IGR, IWMA)[15,17].

Grumeti and Ikorongo Game Reserves – strong protection

Grumeti and Ikorongo Game Reserves (GGR, IGR) are managed jointly between TAWA and Singita Grumeti Limited as one ecological unit with Ikona Wildlife Management Area[1]. Grumeti and Ikorongo Game Reserves do not have an IUCN management category, indicating

that these protected areas have not provided complete information for IUCN designation[3], though Veldhuis et al., (2018) classify them as category II-like (area managed to complement the adjacent national park with similar management). In Tanzania, game reserve status implies that occupation, livestock grazing, and cropland are prohibited, while trophy hunting is permitted[2,13]. However, Singita Grumeti Limited owns all hunting blocks in the area, and prohibits all hunting in GGR and IGR[16,18–21].

Tanzania Wildlife Management Authority provides social services to communities by constructing schools, health centers, and roads[5,19]. Both TAWA and Singita Grumeti Limited provide limited employment opportunities and bushmeat[5,19,22]. Singita Grumeti Limited also pays villages for their hunting rights[16,19], provides partial compensation for crop depredation[13], and provides conservation education[6]. Additionally, a Community Conservation Bank program initiated in 2009 by the Frankfurt Zoological Society in the Serengeti district has helped villagers improve their livelihoods by providing microfinancing to individuals for establishment of environmentally friendly business enterprises[5,6,23,24]. Though villagers have may have contact with Singita Grumeti Limited, much of the contact is hostile and there is little transparency of governance, and little to no community input for decision-making[6,19,25].

Singita Grumeti Limited provides funding for increased law enforcement[2,15,17], including increased numbers of personnel[15,17], establishment of an increased number of permanent ranger camps [26], and provision of vehicles, weapons, and other supplies to law enforcement[19]. Additionally, local people are employed as Village Game Scouts that aid in arresting illegal hunters[19,27]. Because participation in the Community Conservation Bank program requires non-involvement in poaching activities, presence of this program may also reduce poaching levels [23]. Less livestock incursion, tree cutting, landscape burning, and poaching occurs in GGR and IGR

as compared to MGR and SNP[1,2,15,17]. Grumeti and Ikorongo Game Reserves also had higher vegetation productivity (as measured by Normalized Difference Vegetation Index) than MGR and NCA[1,2].

Ikona Wildlife Management Area – strong protection

Ikona Wildlife Management Area (IWMA) is managed under a community-based organization by the surrounding villages of Robanda, Park Nyigoti, Nyichoka, Makundusi, and Nattambiso[28,29]. Ikona Wildlife Management Area does not have an IUCN management category, indicating that it has not provided complete information for IUCN designation[3], though Veldhuis *et al.* (2018) classifies it as category II-like (area managed to complement the adjacent national park with similar management). Ikona Wildlife Management Area prohibits sustainable resource extraction, but the village of Robanda and adjacent surrounding grazing lands are situated in southcentral IWMA but are excluded from the wildlife management area[3,28]. Though hunting is permitted in IWMA, Singita Grumeti Limited reserves the hunting rights in the area and prohibits harvest[19–21].

Ikona Wildlife Management Area uses fifty percent of revenue for conservation and enforcement, and distributes remaining revenue to the five member villages[28,30]. Ikona Wildlife Management Area receives additional support from Singita Grumeti Limited. Financial resources from both organizations are used primarily to improve social infrastructure, including building schools, health centers, and water holes[28,30]. Ikona Wildlife Management Area and Singita Grumeti Limited also provide direct employment opportunities, funding for conservation education, and student scholarships[28,30]. Singita Serengeti Limited provides local communities with a portion of tourism revenue and payment for hunting rights[16,19]. The Community

Conservation Bank program initiated in 2009 by the Frankfurt Zoological Society in the Serengeti district has helped villagers improve their livelihoods by providing microfinancing to individuals for establishment of environmentally friendly business enterprises[5,23,24].

Though only local residents participate in the governance system, there is inadequate communication among community leaders, representatives, and villagers regarding management decisions including investor contracts, disbursement of funds, revenue sharing, and project funding[23,29]. Ikona Wildlife Management Area is among the highest income generating protected areas in Tanzania[1,30], but there is little transparency about funding distribution and households do not receive direct payments from the wildlife management area, and therefore many in the community have negative attitudes of protected areas [23,28,29].

Through collaboration between Singita Grumeti Limited and IWMA, there are high levels of patrols in IWMA[30]. Additionally, local people are employed as Village Game Scouts that aid in arresting illegal hunters[19,27,30]. Because participation in the Community Conservation Bank program requires non-involvement in poaching activities, this program may also reduce extent of poaching[23]. Less livestock incursion, tree cutting, landscape burning, and poaching occurs in IWMA compared to MGR and SNP[1,2].

References

1. Veldhuis, M. P. *et al.* [dataset] Data from: Cross-boundary human impacts compromise the Serengeti-Mara ecosystem. Dryad data repository. (2018) doi:10.5061/dryad.b303788.
2. Veldhuis, M. P. *et al.* Cross-boundary human impacts compromise the Serengeti-Mara ecosystem. *Science* **363**, 1424–1428 (2019).
3. UNEP-WCMC & IUCN. Protected planet: the world database on protected areas (WDPA). www.protectedplanet.net (2020).
4. IUCN. Ngorongoro Conservation Area conservation outlook assessment. *The IUCN World Heritage Outlook* https://worldheritageoutlook.iucn.org/node/964/pdf/en?year=2017 (2020).
5. Kegamba, J. J., Sangha, K. K., Wurm, P. & Garnett, S. T. A review of conservation-related benefit-sharing mechanisms in Tanzania. *Glob. Ecol. Conserv.* **33**, e01955 (2022).
6. Kisingo, A. W. Governance of protected areas in the Serengeti Ecosystem, Tanzania. (University of Victoria, 2013).
7. Melita, A. W. & Mendlinger, S. The impact of tourism revenue on the local communities; livelihood: a case study of Ngorongoro Conservation Area, Tanzania. *J. Serv. Sci. Manag.* **06**, 117–126 (2013).
8. Melubo, K. & Lovelock, B. Living inside a UNESCO World Heritage Site: the perspective of the Maasai community in Tanzania. *Tour. Plan. Dev.* **16**, 197–216 (2019).
9. Maddox, T. M. The ecology of cheetahs and other large carnivores in a pastoralist-dominated buffer zone. (London University College and London Institute of Zoology., 2003).
10. Ikanda, D. & Packer, C. Ritual vs. retaliatory killing of African lions in the Ngorongoro Conservation Area, Tanzania. *Endanger. Species Res.* **6**, 67–74 (2008).
11. Matungwa, L. M. & Wawa, A. I. The effectiveness of anti-poaching techniques in combating wildebeest poaching in Serengeti National Park. **90**, 24–39 (2021).
12. Kideghesho, J. R. Reversing the trend of wildlife crime in Tanzania: challenges and opportunities. *Biodivers. Conserv.* **25**, 427–449 (2016).
13. Schmitt, J. A. Improving conservation efforts in the Serengeti ecosystem, Tanzania: an examination of knowledge, benefits, costs, and attitudes. (University of Minnesota, 2010).
14. Mwampeta, S. B. *et al.* Lion and spotted hyena distributions within a buffer area of the Serengeti-Mara ecosystem. *Sci. Rep.* **11**, 1–8 (2021).
15. Rija, A. A. Spatial pattern of illegal activities and the impact on wildlife populations in protected areas in the Serengeti ecosystem. (University of York, 2017).
16. Kideghesho, J. R. Wildlife conservation and local land use conflicts in Western Serengeti Corridor, Tanzania. (Norwegian University of Science and Technology, 2006).
17. Rija, A. A. & Kideghesho, J. R. Poachers' strategies to surmount anti-poaching efforts in Western Serengeti, Tanzania. in *Protected Areas in Northern Tanzania* (eds. Durrant, J. O. et al.) 91–112 (Springer Nature Switzerland AG, 2020). doi:10.1007/978-3-030-43302-4.
18. Knapp, E. J. Why poaching pays: A summary of risks and benefits illegal hunters face in Western Serengeti, Tanzania. *Trop. Conserv. Sci.* **5**, 434–445 (2012).
19. Zella, A. Y. The management of protected areas in Serengeti ecosystem: a case study of Ikorongo and Grumeti Game Reserves (IGGRs). *Int. J. Eng. Sci.* **6**, 22–50 (2016).
20. Knapp, E. J., Rentsch, D., Schmitt, J., Lewis, C. & Polasky, S. A tale of three villages: choosing an effective method for assessing poaching levels in western Serengeti, Tanzania. *Oryx* **44**, 178–184 (2010).

21. L. Kaswamila, A. & E. Mwakipesile, A. Resident hunting ban in Serengeti District and its implications to people's livelihood and wildlife population. in *Wildlife Population Monitoring* (ed. Ferretti, M.) (IntechOpen, 2019). doi:10.5772/intechopen.83827.
22. Holmern, T., Røskaft, E., Mbaruka, J., Mkama, S. Y. & Muya, J. Uneconomical game cropping in a community-based conservation project outside the Serengeti National Park, Tanzania. *Oryx* **36**, 364–372 (2002).
23. Kaaya, E. & Chapman, M. Micro-credit and community wildlife management: complementary strategies to improve conservation outcomes in Serengeti National Park, Tanzania. *Environ. Manage.* **60**, 464–475 (2017).
24. Sulle, E. B. & Nelson, R. H. The role of microcredit institutions in poverty reduction and on dependence on natural resources A case study of COCOBA in Serengeti District By School of Public Policy , University of Maryland College Park. (2012).
25. Kideghesho, J. R., Røskaft, E. & Kaltenborn, B. P. Factors influencing conservation attitudes of local people in Western Serengeti, Tanzania. *Biodivers. Conserv.* **16**, 2213–2230 (2007).
26. Snyder, K. D., Mneney, P. B. & Wittemyer, G. Predicting the risk of illegal activity and evaluating law enforcement interventions in the western Serengeti. *Conserv. Sci. Pract.* **1**, 1–13 (2019).
27. Holmern, T., Muya, J. & Røskaft, E. Local law enforcement and illegal bushmeat hunting outside the Serengeti National Park, Tanzania. *Environ. Conserv.* **34**, 55–63 (2007).
28. Makupa, E. E. Conservation efforts and local livelihoods in Western Serengeti, Tanzania: experiences from Ikona Community Wildlife Management Area. (University of Victoria, 2013).
29. Kimario, F. F., Botha, N., Kisingo, A. & Job, H. Theory and practice of conservancies: evidence from wildlife management areas in Tanzania. *Erdkunde* **74**, 117–141 (2020).
30. Robinson, L. W. & Makupa, E. Using analysis of governance to unpack community-based conservation: a case study from Tanzania. *Environ. Manage.* **56**, 1214–1227 (2015).
